# Anionic Oligo(ethylene glycol)-Based Molecular Brushes: Thermo- and pH-Responsive Properties

**DOI:** 10.3390/polym16243493

**Published:** 2024-12-14

**Authors:** Alexey Sivokhin, Dmitry Orekhov, Oleg Kazantsev, Ksenia Otopkova, Olga Sivokhina, Ilya Chuzhaykin, Ekaterina Spitsina, Dmitry Barinov

**Affiliations:** 1 Research Laboratory “New Polymeric Materials”, Nizhny Novgorod State Technical University, n.a. R.E. Alekseev, 24 Minin Street, 603155 Nizhny Novgorod, Nizhegorodskaya Oblast, Russia; 2 V.A. Kargin Research Institute of Chemistry and Technology of Polymers with Pilot Plant, 606000 Dzerzhinsk, Nizhegorodskaya Oblast, Russia

**Keywords:** thermo response, pH-responsive molecular brushes, continuous flow, photoiniferter polymerization, self-assembly

## Abstract

Anionic thermo- and pH-responsive copolymers were synthesized by photoiniferter reversible addition–fragmentation chain transfer polymerization (PI-RAFT). The thermo-responsive properties were provided by oligo(ethylene glycol)-based macromonomer units containing hydrophilic and hydrophobic moieties. The pH-responsive properties were enabled by the addition of 5–20 mol% of strong (2-acrylamido-2-methylpropanesulfonic) and weak (methacrylic) acids. Upon initiation by visible light at 470 nm and in the absence of radical initiators, yields from the ternary copolymers reached 94% in 2.5 h when the process was carried out in continuous flow mode using 4-cyano-4-[(dodecylsulfanylthiocarbonyl)sulfanyl]pentanoic acid as a light-sensitive RAFT agent. The polymers were characterized using size exclusion chromatography, IR and NMR spectroscopy, and differential scanning calorimetry. The copolymers featured a sufficiently high molecular weight (93–146 kDa) consistent with theoretical values and satisfactory dispersities in the range of 1.18–1.45. The pH-responsive properties were studied in deionized water, saline, and buffer solutions. Dramatic differences in LCST behavior were observed in strong and weak acid-based polyelectrolytes. The introduction of sulfonic acid units, even in very small amounts, completely suppressed the LCST transition in deionized water while maintaining it in the saline and buffer solutions, with a negligible LCST dependence on the pH. In contrast, the incorporation of weak methacrylic acid demonstrated a pronounced pH dependence. The peculiarities of micelle formation in aqueous solutions were investigated and critical micelle concentrations and their ability to retain pyrene, a hydrophobic drug model, were determined. It was observed that anionic molecular brushes formed small micelles with aggregation numbers of 1–2 at concentrations in the order of 10^−4^ mg/mL. These micelles have a high ability to entrap pyrene, which makes them a promising tool for targeted drug delivery.

## 1. Introduction

In recent decades, many effective pharmaceutical substances (PSs) have been developed to treat various diseases. However, these substances often exhibit adverse effects on the human body at high doses. To optimize treatment outcomes, it is essential to maintain a stable and optimal concentration of PSs in target organs over an extended period. This can be achieved through controlled delivery systems, which enable a sustained release of PSs while minimizing their toxicity [1]. One promising approach is the development of nanopolymeric carriers, which can selectively deliver PSs to target sites while reducing their accumulation in healthy tissues [2,3]. Polymeric nanoparticles containing oligo(ethylene glycol) moieties have emerged as a versatile platform for controlled drug delivery, owing to their unique properties, such as biocompatibility, biodegradability, and their ability to solubilize poorly-water-soluble compounds [4,5,6,7]. Methoxy oligo(ethylene glycol) methacrylate (MOEGMA) copolymers have garnered significant attention as potential nanocarriers for PS delivery [8,9,10]. These molecular brushes exhibit unique self-organization properties, allowing them to form regular structures in a solution [11,12,13,14]. The incorporation of hydrophobic alkyl fragments into MOEGMA copolymers enables the formation of stable, unimolecular micelles with a narrow size distribution, which can effectively solubilize low-polarity compounds [15,16,17]. The critical micelle concentration (CMC) of these copolymers can be easily regulated by varying the length of the oligo(ethylene glycol) (OEG) chains, allowing for precise control over the assembly and disassembly of the micellar structures [18,19]. The ability of these polymers to form stable, unimolecular micelles in water is attributed to steric repulsion of the densely grafted OEG chains, which prevents intermolecular aggregation [14].

To further tailor the properties of polymeric nanocarriers, researchers have explored the incorporation of pH-responsive units, such as carboxylic, amino or ammonium groups, which can respond to changes in their environmental pH [20,21,22,23,24]. This enables the development of smart delivery systems that can release PSs in response to specific stimuli. Overall, the development of polymeric nanocarriers offers a promising approach to controlled PS delivery and ongoing research in this field is expected to yield novel materials with properties tailored for improved treatment outcomes. The versatility of these polymeric systems, combined with their ability to respond to specific stimuli, makes them an attractive platform for the development of smart delivery systems that can revolutionize the field of pharmaceuticals [25,26].

The incorporation of thermo- and pH-responsive units can be achieved through various methods, including grafting, copolymerization, or cross-linking [27]. The synthesis of molecular brushes most commonly involves the use of controlled radical polymerization techniques, such as reversible addition–fragmentation chain transfer (RAFT) polymerization, which enables precise control over polymer structure and composition [15,19,28].

In the present work, PI-RAFT polymerization has been applied to the synthesis of molecular brushes. The advantages of PI-RAFT include the use of low-cost visible light and ambient reaction conditions. This method enables the efficient production of block copolymers with spatiotemporal control and high chain-end fidelity [29,30,31,32,33,34,35,36,37,38,39,40]. PI-RAFT is a popular approach in which the chain transfer agent functions as an initiator, generating free radicals when exposed to visible light. Notably, photoiniferter polymerization has no tolerance for oxygen [41]. However, using a continuous photoreactor in conjunction with a suitable solvent enables high conversions to be achieved in a short timeframe (2–3 h) while ensuring an acceptable level of control [42,43]. This approach also offers the advantage of scalability. A number of investigations have revealed that continuous flow reactors offer significant advantages compared to traditional periodic processes in photocatalytic radical polymerization [44,45,46,47,48].

The present study is focused on the synthesis of ternary amphiphilic thermo- and pH-responsive molecular brushes. The base that provides these molecular brushes with their thermo-sensitive properties consists of methoxy and alkoxy oligo(ethylene glycol) methacrylate units as well as modifies acidic comonomers, including 2-acrylamido-2-methylpropanesulfonic acid and methacrylic acid, to impart pH-responsive properties. The objective of this research is to explore the formation mechanisms of these copolymers and their self-assembly in aqueous solutions as well as gain insight into the impact of acidic units on these processes.

## 2. Materials and Methods

### 2.1. Materials

**Figure 1:**
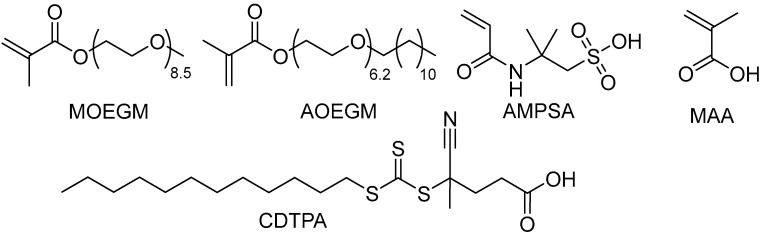
Starting materials used for molecular brush preparation.

Alkoxy (C_12_-C_14_) oligo(ethylene glycol) methacrylate (AOEGM) and oligo(ethylene glycol) methyl ether methacrylate (MOEGM, Mn = 500) from Sigma-Aldrich (Moscow, Russia) (Figure 1) were used as base macromonomers, which were purified of inhibitors by passing them through a column filled with basic alumina. AOEGM was synthesized by the esterification of methacrylic acid with alkoxy oligo(ethylene glycol) containing a C_12_-C_14_ alkyl fragment (Zavod sintanolov LLC, Dzerzhinsk, Russia) in the presence of p-toluene sulfonic acid (JSC Khimreaktiv, Nizhny Novgorod, Russia) as a catalyst. As acidic comonomers, 2-Acrylamido-2-methylpropanesulfonic acid, AMPSA, without additional purification from Sigma-Aldrich (Moscow, Russia) and methacrylic acid (JSC Khimreaktiv, Nizhny Novgorod, Russia) purified by distillation were used. The RAFT agent (4-cyano-4-[(dodecylsulfanylthiocarbonyl)sulfanyl]pentanoic acid), CDTPA, was synthesized according to the procedure described in the work [49]. Other reagents, namely, acetonitrile (for spectroscopy, ≥99.5%), tetrahydrofuran (ACS reagent, ≥99.5%), and dimethyl sulfoxide (ACS reagent, ≥99.5%) from Aldrich (Moscow, Russia) were used as received.

### 2.2. Synthesis of Anionic Molecular Brushes

**Scheme 1:**
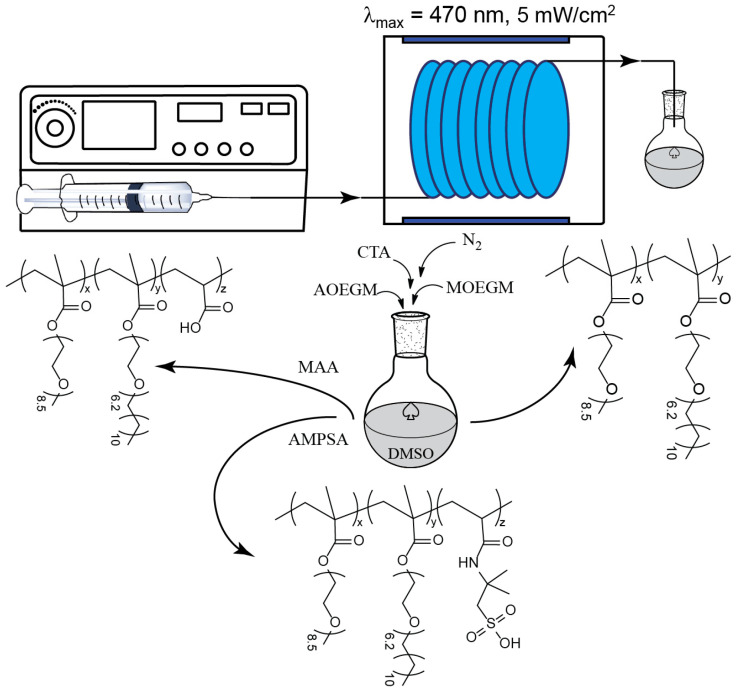
Schematic diagram for molecular brush synthesis.

The flow reactor for PI-RAFT polymerization consisted of a glass cylinder with a diameter of 7.5 cm attached to a PTFE tube with an inner diameter of 2 mm, a wall thickness of 1 mm, a total working length of 5.9 m, and a volume of 18.5 cm^3^. The outside of the reaction tube was uniformly illuminated by visible light with the wavelength λ_max_ = 470 nm, generated using a household LED strip (Scheme 1). The light intensity near the reaction volume was controlled by a PS3005N switch mode power supply from QJE (Xinyujie Electronics Co., Ltd., Shenzhen, China) at 5 mW/cm^2^. An OHSP-350C spectral analyzer (Hangzhou Hopoo Light&Color Technology Co., Ltd., Hangzhou, China) was used to measure light intensity.

The procedure for the preparation of anionic molecular brushes was the following: CDTPA (12.6 mg, 30.3 μmol, 1.0 eq.) was dissolved in DMSO (3.16 g). AMPSA (0.1155 g, 0.55 mmol, 18.2 eq.), oligo(ethylene glycol) methyl ether methacrylate (0.8940 g, 1.93 mmol, 64 eq.), and alkoxy oligo(ethylene glycol) methacrylate (2.1566 g, 3.55 mmol, 118 eq.) were added to the resulting solution. The mixture was stirred until homogenized, purged with nitrogen to remove dissolved oxygen, and injected at a specified rate into a tubular reactor using a syringe pump. The syringe with the initial mixture and the receiving vial were protected from light by foil. The final concentration of monomers was 50 wt%. The resulting reaction mixtures were diluted with 3–5 times ethyl alcohol and purified by dialysis (MWCO 8–14k) against ethyl alcohol with a 4-time solvent exchange. The resulting solution was concentrated on a rotary evaporator and dried at 5 mmHg to a constant weight. The copolymers were very viscous (honey-like) substances, yellowish in color (due to CTA residues). The structures were confirmed by IR and ^1^H NMR spectroscopy (Supplementary Materials, Figures S1–S8). The number-average molecular weights were determined, where possible, from the RAFT agent terminal group signals.

### 2.3. Characterization Techniques

#### 2.3.1. Size Exclusion Chromatography

A Chromos LC-301 system with an Alpha-10 isocratic pump and a Waters 410 refractometer detector (Chromos, Dzerzhinsk, Russia) was used to determine the molecular weights and dispersities of the ternary copolymers. Two exclusive Phenogel 5 µm 500A and Phenogel 5 µm 10E5A columns from Phenomenex (measurement range of 1–1000 kDa) were used, with tetrahydrofuran as the eluent.

#### 2.3.2. Differential Scanning Calorimetry

The polymer samples (approximately 10–15 mg) were subjected to differential scanning calorimetry (DSC) analysis in an aluminum pan under a dry argon flow. The experiments were conducted using a DSC 204F1 Phoenix calorimeter (Netzsch, Selb, Germany) with CC 200 liquid nitrogen cooling controller. Heating and cooling rates of 10 and −10 °C per minute, respectively, were applied between −80 and 80 °C.

#### 2.3.3. NMR Spectroscopy

One-dimensional proton nuclear magnetic resonance spectra were acquired at ambient temperature in deuterated chloroform using an Agilent DD2-400 MHz spectrometer (Agilent Technologies, Santa Clara, CA, USA). The chemical shifts were calibrated using residual resonances from the solvent, which served as a reference point for calibration purposes.

#### 2.3.4. Static Light Scattering (SLS)

Light scattering experiments were conducted on a Photocor Complex instrument (Photocor LLC, Moscow, Russia) with a goniometer and a thermostabilized diode laser (λ = 659 nm, 35 mW). Temperature was maintained by a thermoelectric Peltier temperature controller, which had a temperature range of 5 to 100 °C and an accuracy of 0.1 °C.

The polymer solutions were agitated for 24 h at a temperature below the lower critical solution temperature (LCST) to achieve equilibrium and filtered using CHROMAFIL PET syringe filters (MACHEREY-NAGEL GmbH & Co., KG, Düren, Germany) with a pore size of 0.20 μm. The weight-average molecular weight (Mw) and the second virial coefficient (A_2_) were determined using the Debye method, given there was no angular dependence of the scattered light intensity.

The configuration employed involved a vertically polarized incident light and detection without a polarizer (VU, Rv geometry). In accordance with the findings of [50], the Rayleigh coefficient for toluene at a wavelength of 659 nm and a measurement temperature of 25 °C was determined to be 1.142 × 10^−5^ cm^−1^ at 25 °C.

The dn/dc values for the copolymers were determined at 30 °C using a BI-DNDC differential refractometer from Brookhaven Instruments Corp. from Holtsville, NY, USA.

#### 2.3.5. Fluorescence Spectroscopy

Fluorescence spectroscopy was employed to ascertain the critical micelle concentrations of the copolymers in aqueous solutions. The copolymer solutions were prepared by solubilizing the samples in an aqueous pyrene solution (6 × 10^−7^ M) to obtain concentrations ranging from 10^−6^ to 0.5 mg/mL. Subsequently, the resulting mixtures were subjected to sonication for five minutes then stored at room temperature for 24 h prior to measurement. The steady-state fluorescence spectra were obtained using a Shimadzu RF-6000 spectrofluorometer (Shimadzu, Tokyo, Japan) under the following conditions: a slit width for excitation of 3 nm, a slit width for emission of 3 nm, a scan speed of 200 nm/min, an excitation wavelength of 335.0 nm, and an emission wavelength range of 350.0–500.0 nm. The intensity ratios of the first (I_1_, 373 nm) and third (I_3_, 384 nm) vibronic pyrene emission bands, designated (I_1_/I_3_), were determined as a function of copolymer concentration. The inflection point on concentration dependence was considered the critical micelle concentration.

#### 2.3.6. Drug Loading Capacity

A model of the capacity of polymer micelles to encapsulate hydrophobic drugs was evaluated using UV spectroscopy. Five milligrams of dried pyrene (the model) was added to a ten milliliter 0.1% (*w*/*v*) aqueous solution of the polymer. The resulting mixture was subjected to sonication for 30 min at 15–25 °C. A syringe filter with a pore size of 450 nm was used to filter the solution containing both pyrene and polymer. The resulting filtrate was dissolved 40-fold in acetonitrile. The pyrene concentration was determined using a Shimadzu UV-1800 spectrophotometer (Shimadzu, Tokyo, Japan) at an absorbance wavelength of 334 nm. A calibration curve was constructed using pyrene standards in acetonitrile.

## 3. Results and Discussion

### 3.1. Synthesis and Characterization of Anionic Molecular Brushes

This study aimed to obtain thermo- and pH-responsive anionic molecular brushes that could form micelles in aqueous solutions and retain hydrophobic drugs. Building on the known thermo-responsive properties of MOEGM–AOEGM copolymers [16,51], we designed an additional copolymer backbone comprising acidic comonomers to assess its impact on self-assembly behavior, critical micelle concentration, and drug loading capacity. We introduced two types of modifying comonomer units, strongly acidic (AMPSA) and weakly acidic (MA), to assess their contribution to pH-responsive properties. Previously, we identified the conditions for the synthesis of molecular brushes of the required composition and molecular weight to form monomolecular micelles in aqueous solutions. Using a combination of methods, including SEC, photon correlation spectroscopy, and static light scattering, we determined the threshold value for the degree of polymerization at which the molecular brushes form single-chain micelles. Below this limit, the macromolecules aggregate to form micelles containing up to 20 chains. This aggregation occurred even before the LCST, and the solutions remained homogeneous, whereas above the LCST, a classical phase transition with polymer separation into a distinct phase was observed [52].

To produce self-assembled copolymers with high chain-end fidelity, we developed a methodology using periodic PI-RAFT polymerization. However, scaling up the synthesis process proved challenging, with polymerization either not occurring or exhibiting a long induction period in reaction vessels larger than 5–10 mL (at the radiation intensity used), resulting in low monomer conversions. A flow reactor was used to address this problem, enabling us to obtain copolymers with a high molecular weight, narrow molecular weight distribution, and a nearly quantifiable yield in 2–2.5 h.

**Figure 2:**
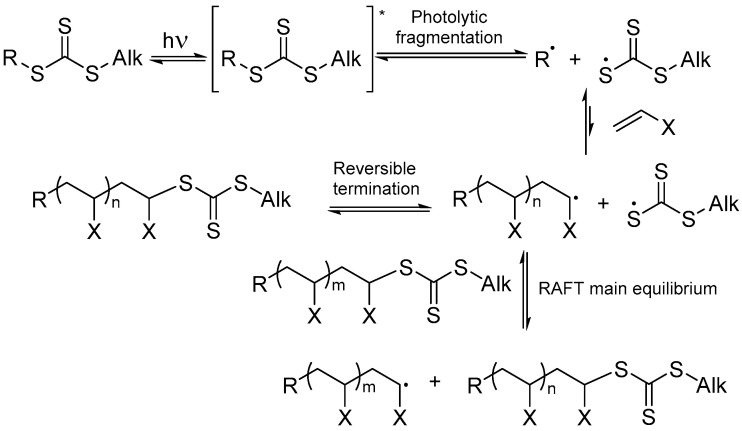
Proposed mechanism of PI-RAFT polymerization.

The copolymers were prepared using light-sensitive CTA, specifically 4-cyano-4-[(dodecylsulfanylthiocarbonyl)sulfanyl]pentanoic acid, decomposed under blue light. The proposed mechanism for the PI-RAFT process involved a trithiocarbonate chain-transfer agent initiated by visible light and is illustrated in Figure 2.

In the case of the PI-RAFT process, polymerization is initiated by homolytic cleavage of the C-S bond, resulting in the elimination of the leaving group. Either π-π* or n-π* transitions are responsible for driving this process. The band associated with photo-excitation through the n-π* transition is weaker, but produces less side reactions of CTA degradation. The required wavelength varies depending on the CTA type, falling within the visible region for trithiocarbonates and dithiobenzoates [53].

**Figure 3:**
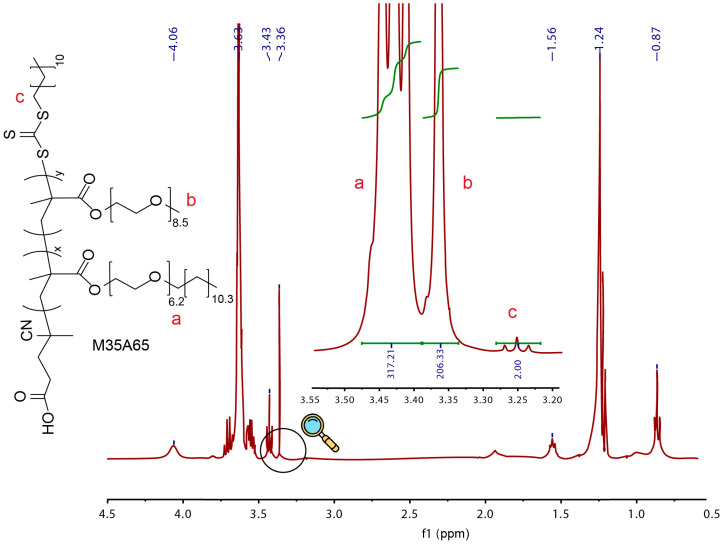
The NMR spectrum of the copolymer. The enlarged region contains signals of the terminal methyl group of MOEGM, the methylene group of the alkyl moiety of AOEGM, and the methylene group of the RAFT agent to establish the molecular weight.

The results of the polymerizations are presented in Table 1. For convenience, the samples are labeled according to the composition of the initial monomer mixture. Achieving the highest possible monomer conversion while keeping dispersity within acceptable limits was our primary goal. Maximum conversions were achieved in samples higher in MOEGMA content relative to AOEGMA. Notably, the theoretical molecular weights and SEC data show a significant inconsistency, the latter markedly underestimated. This has already been reported in bottlebrushes containing oligo(ethylene glycol) units [16,19,54,55,56], except for those with a low molecular weight [35,44,57]. We believe the major reason for this inconsistency is likely the nonlinear dependence of residence time on molecular weight in SEC caused by the macromolecular shape and change in hydrodynamic volume as the degree of polymerization increased. Molecular brushes, unlike linear polymers, transform their conformation from a spherical to a worm-like structure with growing degrees of polymerization. Therefore, where possible, Mn was determined by ^1^H NMR spectroscopy using the signal of the RAFT agent methylene protons adjacent to sulfur (triplet at 3.25 ppm, peak c in Figure 3). Absolute molecular weight data are summarized in Table 1.

**Figure 4:**
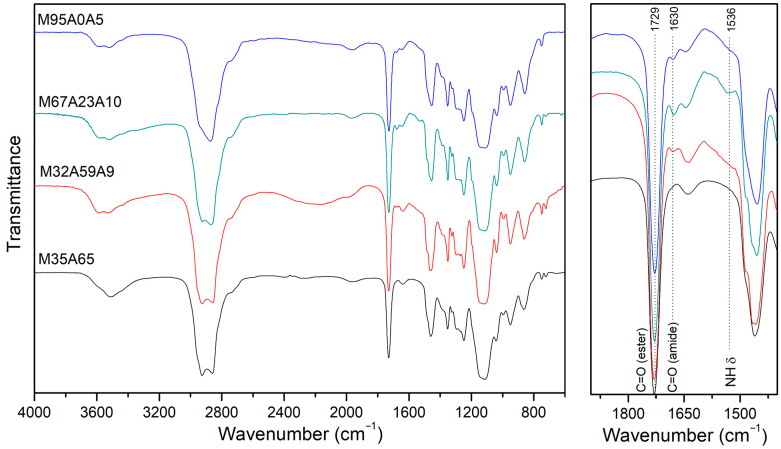
The IR spectra of AMPSA-containing molecular brushes. Sample designations are shown in Table 1. The enlarged region shows the carbonyl signals of the ester and amide groups, as well as a poorly visible shoulder associated with the δNH vibrations of the AMPSA amide group.

Incorporation of the AMPSA comonomer is confirmed by infrared (IR) spectroscopy data, as shown in Figure 4. The IR spectrum of the molecular brushes containing AMPSA shows characteristic bands assigned to the carbonyl group stretching vibration (C=O, 1729 cm^−1^), ether bond asymmetric stretching vibration (C-O-C, 1115 cm^−1^), and bands of methylene (-CH_2_) and methyl (-CH_3_) groups at 2861 cm^−1^ and 2925 cm^−1^, respectively. The broad band at 3300–3700 cm^−1^ is attributed to OH vibrations in hydrogen-bonded water. The absorption bands at 1678 and 1536 are attributed to C=O (amide) stretching and NH bending vibrations, respectively. It should be noted the significantly lower activity of acrylamides in copolymerization with methacrylates; a similar situation is typical of acrylamido–sulfonic acids, including AMPSA. The amide carbonyl peaks indicate very low AMPSA content in the copolymers.

**Figure 5:**
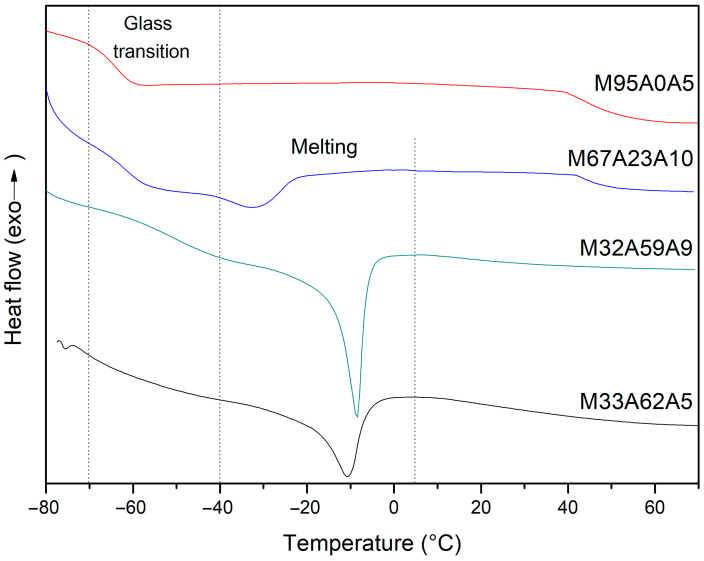
DSC thermograms for p(MPEGMA-AOEGM-AMPSA)s. Heating rate: 10 °C/min.

The copolymers exhibited two distinct types of transition in the DSC thermograms, namely, glass transition and melting. The first transition is due to MOEGMA units and the second is due to the melting of alkyl fragments of AOEGMA. Table 2 presents the numerical values of Tg and Tm. The glass transition becomes more pronounced in samples with a higher content of MOEGM, M67A23A10, and M95A0A5, whereas the transition is hardly noticeable in samples of about 33% content. The glass transition temperature varied, ranging from −48 to −64 °C, depending on the copolymer composition. The melting point (Tm) of AOEGMA was observed to broaden in all the copolymers as a consequence of the presence of OEG moieties, which are inherently poorly crystallizable (Figure 5).

### 3.2. Thermo- and pH-Responsive Properties

It has previously been established [52] that MOEGM-AOEGM-based copolymers demonstrate thermo-responsive behavior, allowing for the precise control of the LCST in a broad range by tuning the proportion of hydrophobic and hydrophilic units. Small amounts of acidic comonomers were additionally introduced to impart pH-responsive properties. Of interest were how they would affect thermo-responsive behavior and how electrolyte strength would affect pH-responsivity.

**Figure 6:**
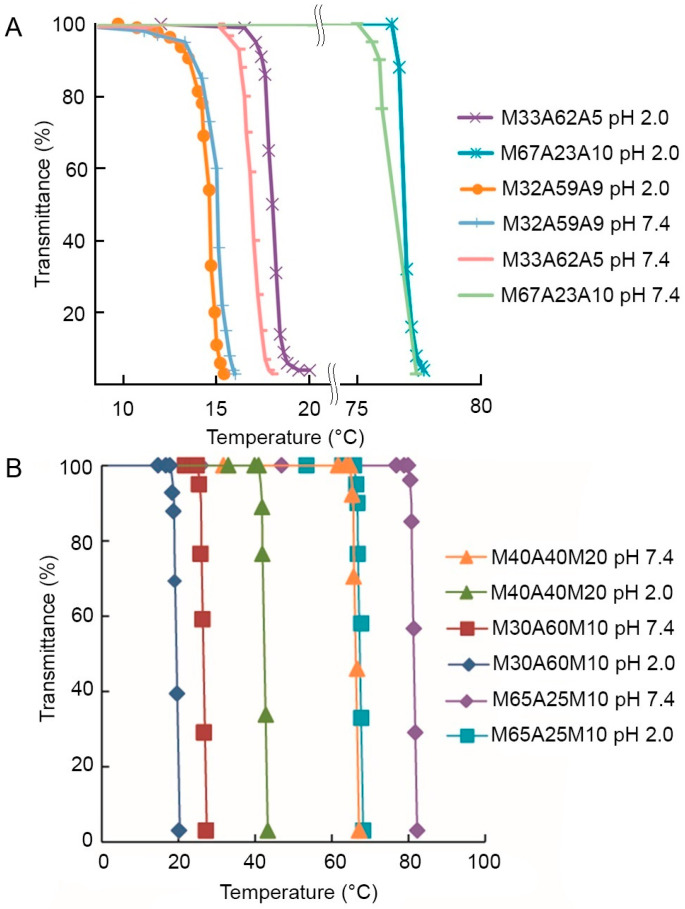
Turbidity curves at different pHs for copolymers containing AMPSA (**A**) and MAA (**B**).

In particular, it should be noted that sample M35A65, chosen for comparison and containing no ionogenic units, had a pronounced LCST transition at about 32 °C in deionized water; the introduction of up to 5% sulfonic acid units completely suppressed the thermo-responsive properties in deionized water and there was no phase separation in all the sulfonic acid polyelectrolyte samples. At the same time, in the buffer mixtures, LCST transition was pronounced. The light transmittance curves of copolymers containing sulfonic acid and carboxylic acid units are presented in Figure 6A,B. All the samples, except for copolymers not containing hydrophobic macromonomer units (AOEGM), tended to demonstrate a thermo-responsive transition at very narrow temperature intervals, even at high molecular weights. This indicates good polydispersity and high compositional homogeneity in copolymers ensured by RAFT polymerization; otherwise, thermo-responsive transitions would be greatly stretched. The MOEGM–AMPSA copolymer (M95A0A5) was too hydrophilic and did not exhibit LCST behavior.

As for the pH-responsive properties in samples containing strong electrolyte units (AMPSA), they are very weakly expressed: In the pH range of 2–7, the phase transition temperature changes, at most, by 1 °C (in the M32A59A9 sample). In the pH range of 7–9, the effect reaches 5 °C in the same sample.

A completely different picture is observed in copolymers containing weak electrolyte MA units; in these cases, the shift in phase transition temperature when changing from pH 2 to 7 can reach 24 °C (as in sample M40A40M20, which contained the maximum percentage of MA units and had an equimolar ratio of base comonomers). Sample M40A40M20 showed a prolonged transition at pH = 9.18 within a 40–90 °C range, with transmittance at the last point of slightly less than 60%.

For all the ternary copolymers, as the fraction of hydrophobic comonomer increases, the LCST consistently decreases. The basis of pH-responsivity in the copolymers is the effect of a decrease in the electrostatic repulsion of the units as the dissociation degree of ionogenic groups decreases. Therefore, the manifestation of pH-responsive properties is greater in cases of weak electrolytes whose degree of dissociation is greatly dependent upon pH, and is practically absent in cases of highly acidic AMPSA units, which are dissociated almost entirely at an acidic pH, seemingly even in the copolymer.

However, the saline nature of the buffer solutions also has a significant influence on thermo-responsive properties, likely due to a hydrophobic effect (“salting out”). This was found when NaCl was introduced; the phase transition temperature shifted significantly to a low value region. For example, in a copolymer containing no ionogenic groups (M35A65), the LCST shifted from 33 °C in pure water to 22 °C in 0.1 M NaCl. At the same time, copolymers containing AMPSA units, which did not exhibit LCST behavior in pure water, did exhibit it in saline solutions (see Table 3).

### 3.3. Micellization and Pyrene Loading Capacity

Determination of critical micelle concentrations in the copolymers was performed using pyrene as a fluorescent probe. In addition, pyrene was used to model a water-insoluble drug, thus enabling the evaluation of the drug loading capacity of the micelles. Overall, the molecular brushes exhibited very low critical micelle concentrations and high loading capacity values, which can be attributed to their amphiphilic nature. The data on CMC and pyrene loading capacity for sulfonic acid molecular brushes are presented in Table 4.

As the fraction of hydrophobic comonomer (AOEGMA) increased, the CMC in the polymeric micelles naturally decreased. As for loading capacity, the presence of acidic units had practically no influence on the involvement of pyrene in macromolecular micelles due to the absence of hydrogen-bonding centers and electrostatic interaction centers in the pyrene molecule. Thus, the loading capacity of pyrene was determined solely by its hydrophobic unit content. The situation may change significantly when using real drugs containing polar groups.

It is logical that the most hydrophilic sample absorbed the lowest amount of pyrene, and since the latter was still in a rather hydrophilic region, the method used to determine CMC was not quite suitable in this case (there was no clear inflection point on the concentration dependence). Therefore, the CMC for M95A0A5 was not determined, but this does not mean that the copolymer did not form micelles or other aggregates. The hydrophobic chain could also form a hydrophobic micelle core or aggregation could occur due to hydrogen bonding or electrostatic interactions.

### 3.4. Molecular Weight Characteristics of Bottlebrushes

The nature of monomers and the ratio of hydrophobic and hydrophilic units in a macromolecule, as well as the length and density of side chains, are the most important parameters in the fine-tuning of amphiphilic properties and LCST. By optimizing the hydrophilic/hydrophobic balance and molecular weight of the copolymers within a certain range, it is possible to obtain molecular brushes capable of forming single-chain micelles in water. Depending on the flexibility of the backbone and the length and amphiphilicity of the side chains, such micelles may be a cylindrical or classical spherical shape. In the latter case, the micelle core may consist of a backbone and hydrophobic side chains directed inside the micelle, while the hydrophilic side chains are located outside.

Moreover, the self-folding effect is possible only when a certain degree of polymerization is reached, depending on the flexibility of the backbone chain. Copolymers of the exact same composition but with a low molecular weight are incapable of self-folding and form multimolecular micelles to reduce the contact surface of the hydrophobic units with water. Previously [51], in the acid-free analogues of the synthesized molecular brushes, we have demonstrated this self-folding ability in aqueous solutions when a certain degree of polymerization is reached (approximately 280–290, which corresponds to a molecular weight of 140 kDa).

In this work, we attempted to demonstrate the influence of strong and weak acid units introduced in minor proportions, the capacity for intermolecular interaction through hydrogen bonding and dipole–dipole interaction on the micelle aggregation number, and the possibility of unimolecular micelle preparation. The number of chains in the micelle (or aggregation number) in water is estimated by analyzing the aqueous solutions using the static light scattering method, which provides information about the average molecular weight of aggregates, if such a process takes place. Unlike the SEC and NMR methods, static light scattering does not provide information about the absolute molecular weight of macromolecules. To calculate the number of chains in the aggregate of the aqueous solutions, we used Equation (1) below. The molecular weight of the polymer aggregates in water (Mw) is divided by the absolute molecular weight of the copolymers (Mn), determined by ^1^H NMR and multiplied by the dispersity, Đ, which is determined by the SEC.

N_agg_ = M_W_(SLS in water)/M_n_(^1^H NMR) ∙ Đ(SEC) (1)

Table 5 summarizes the molecular weight characteristics of the anionic sulfonic acid molecular brushes. All the investigated copolymers showed an average aggregation number in water not exceeding two, indicating the predominant formation of mono- and bi-molecular micelles in aqueous solutions. Indeed, all the samples, except M67A23A10, had an absolute molecular weight, Mn, close to the threshold (140 kDa). Exceeding this value, the latter indicated the presence of macromolecules in water with an aggregation number close to unity.

## 4. Conclusions

Anionic thermo- and pH-responsive molecular brushes consisted of oligo(ethylene glycol)-containing macromonomers and up to 20% of strong and weak acid comonomers were successfully synthesized by photoiniferter polymerization in a continuous photoreactor. The molecular brushes featured a satisfactory dispersity of 1.18–1.45 and high yields reaching 94% in 2.5 h.

The copolymers were characterized using differential scanning calorimetry, static light scattering, and turbidimetry. The thermal analysis showed that the copolymers exhibited glass transition temperatures in the range of approximately −48 to −64 °C due to the presence of MOEGM units, and melting points in the range of −33 to −8 °C due to the melting of AOEGM alkyl fragments.

It has been demonstrated that MOEGM–AOEGM copolymers display thermo-responsive behavior, offering the potential for precise adjustment of lower critical solution temperature across a broad range by altering the ratio of hydrophilic (MOEGM) and hydrophobic (AOEGM) units. Small amounts of acidic comonomers were introduced to impart pH-responsive properties. It was shown that the introduction of up to 5% sulfonic acid units completely suppressed thermo-responsive properties in deionized water; phase separation was not observed. Conversely, in the buffer mixtures, the LCST transition was pronounced.

In samples containing strong electrolyte units (AMPSA), pH-responsivity was extremely weak, with a maximum change of 5 °C in the phase transition temperature within a pH range of 2–9. In contrast, copolymers comprising a weak acid, MA, exhibited a significant shift, reaching as much as 24 °C when the pH was altered from 2 to 7 in the sample with the highest percentage of MA units. As the proportion of hydrophobic comonomers in all the ternary copolymers increased, the lower critical solution temperature consistently decreased. It has been shown that the salinity of buffer solutions has a significant effect on thermo-responsive properties, likely due to their hydrophobic effect (“salting out”). The introduction of NaCl led to a significant shift in phase transition temperature toward a low temperature region.

Anionic molecular brushes have demonstrated extremely low critical micelle concentrations due to their amphiphilic nature and high loading capacity for pyrene.

## Figures and Tables

**Table 1:** Characterization of (co)polymers.

ID	[MOEGM]_0_ /[AOEGM]_0_ /[M]_0_	Comonomer (M)	Flow Rate, mL/min	Time, min	Conversion, %	M_n,th_	M_n_	M_w_	Đ (M_w_/M_n_)
M35A65	35:65:0	-	9	128	89	96,200	12,550	16,100	1.28
M33A62A5	33.4:61.9:4.7	AMPSA	8	135	84	88,650	11,800	16,200	1.37
M32A59A9	31.8:59.1:9.1	AMPSA	8	134	79	83,050	12,600	17,500	1.38
M67A23A10	67.5:22.5:10	AMPSA	8	140	86	81,900	4950	5850	1.18
M95A0A5	95:0:5	AMPSA	9	130	92	84,300	2200	3000	1.40
M30A60M10	30:60:10	MA	7	150	90	84,800	3200	4640	1.45
M40A40M20	40:40:20	MA	8	138	90	79,000	13,200	18,200	1.37
M65A25M10	65:25:10	MA	7	150	94	81,100	4850	6250	1.29

**Table 2:** Thermal characterization of molecular brushes.

Sample ID	Tg, °C	Tm, °C
M33A62A5	Absent/undefined	−10.9
M32A59A9	−48.3	−8.5
M67A23A10	−60.5	−32.7
M95A0A5	−63.9	Absent

**Table 3:** Thermo- and pH-responsive properties of anionic molecular brushes (copolymer concentration of 10 mg/mL).

ID	LCST, °C			
	pH 2.0	pH 7.4	pH 9.18	0.1M NaCl
M35A65	-	-	-	22.0
M33A62A5	18.0	16.9	19.5	18.3
M32A59A9	14.6	15.1	20.6	14.2
M67A23A10	76.7	76.8	>93 (or absent)	-
M95A0A5	>93 (or absent)	>93 (or absent)	>93 (or absent)	86.5
M30A60M10	42.7	66.2	>93 (or absent)	-
M40A40M20	19.7	26.7	40–90 (τ < 60%)	-
M65A25M10	67.0	80.4	>93 (or absent)	-

**Table 4:** Critical micelle concentrations and loading capacity of molecular brushes.

Sample ID	CMC (mg/mL)	Loading Capacity (mg Pyrene/g Polymer)
M35A65	1.9 × 10^−3^	27.0
M33A62A5	7.2 × 10^−4^	28.3
M32A59A9	6.7 × 10^−4^	31.3
M67A23A10	1.55 × 10^−3^	11.3
M95A0A5	Absent/undefined	1.1

**Table 5:** Absolute molecular weights of macromolecules (Mn) and their aggregates in water (Mw).

ID	M_n_	$M_{{W,H}_{2}O}$	$A_{{2,H}_{2}O}\cdot 10^{4},$ mole·cm^3^·g^−2^	N_agg_ in Water
M35A65	116,500	288,900	−0.18	1.9
M33A62A5	94,300	251,500	1.14	1.9
M32A59A9	93,600	204,000	0.00	1.6
M67A23A10	145,650	112,600	9.27	0.7
M95A0A5	-	210,100	1.35	1.8

## Data Availability

The original contributions presented in this study are included in the article/Supplementary Materials. Further inquiries can be directed to the corresponding authors.

## Supplementary Materials

The following supporting information can be downloaded at: https://www.mdpi.com/article/10.3390/polym16243493/s1, Figure S1: ^1^H NMR spectrum of M35A65 copolymer in CDCl_3_; Figure S2: ^1^H NMR spectrum of M33A62A5 copolymer in CDCl_3_; Figure S3: ^1^H NMR spectrum of M32A59A9 copolymer in CDCl_3_; Figure S4: ^1^H NMR spectrum of M67A23A10 copolymer in CDCl_3_; Figure S5: ^1^H NMR spectrum of M95A0A5 copolymer in CDCl_3_; Figure S6: ^1^H NMR spectrum of M30A60M10 copolymer in CDCl_3_; Figure S7: ^1^H NMR spectrum of M40A40M20 copolymer in CDCl_3_; Figure S8: ^1^H NMR spectrum of M65A25M10 copolymer in CDCl_3_.
