# Supplementary material for: Anionic Oligo(ethylene glycol)-Based Molecular Brushes: Thermo- and pH-Responsive Properties
Source: Polymers (Basel). 2024 Dec 14;16(24):3493. doi: 10.3390/polym16243493 (PMC11728562; doi:10.3390/polym16243493)
Supplement: Supplementary file 1 [file polymers-16-03493-s001.zip › polymers-3360109-supplementary.pdf]

Supplementary Information for:

Anionic oligo(ethylene glycol)-based molecular brushes: thermo- and pH-responsive properties

Alexey Sivokhin [1,*], Dmitry Orekhov [1], Oleg Kazantsev [1], Ksenia Otopkova [1], Olga Sivokhina [2], Ilya Chuzhaykin [1], Ekaterina Spitsina [1], Dmitry Barinov [1]

[1] Research Laboratory "New Polymeric Materials", Nizhny Novgorod State Technical University n.a. R.E. Alekseev, 24 Minin Street, 603155 Nizhny Novgorod, Russia
[2] V.A. Kargin Research Institute of Chemistry and Technology of Polymers with Pilot Plant, 606000 Dzerzhinsk, Nizhegorodskaya obl., Russia
*Correspondence: sivokhin@dpingtu.ru; Tel.: +7-831-334-7166

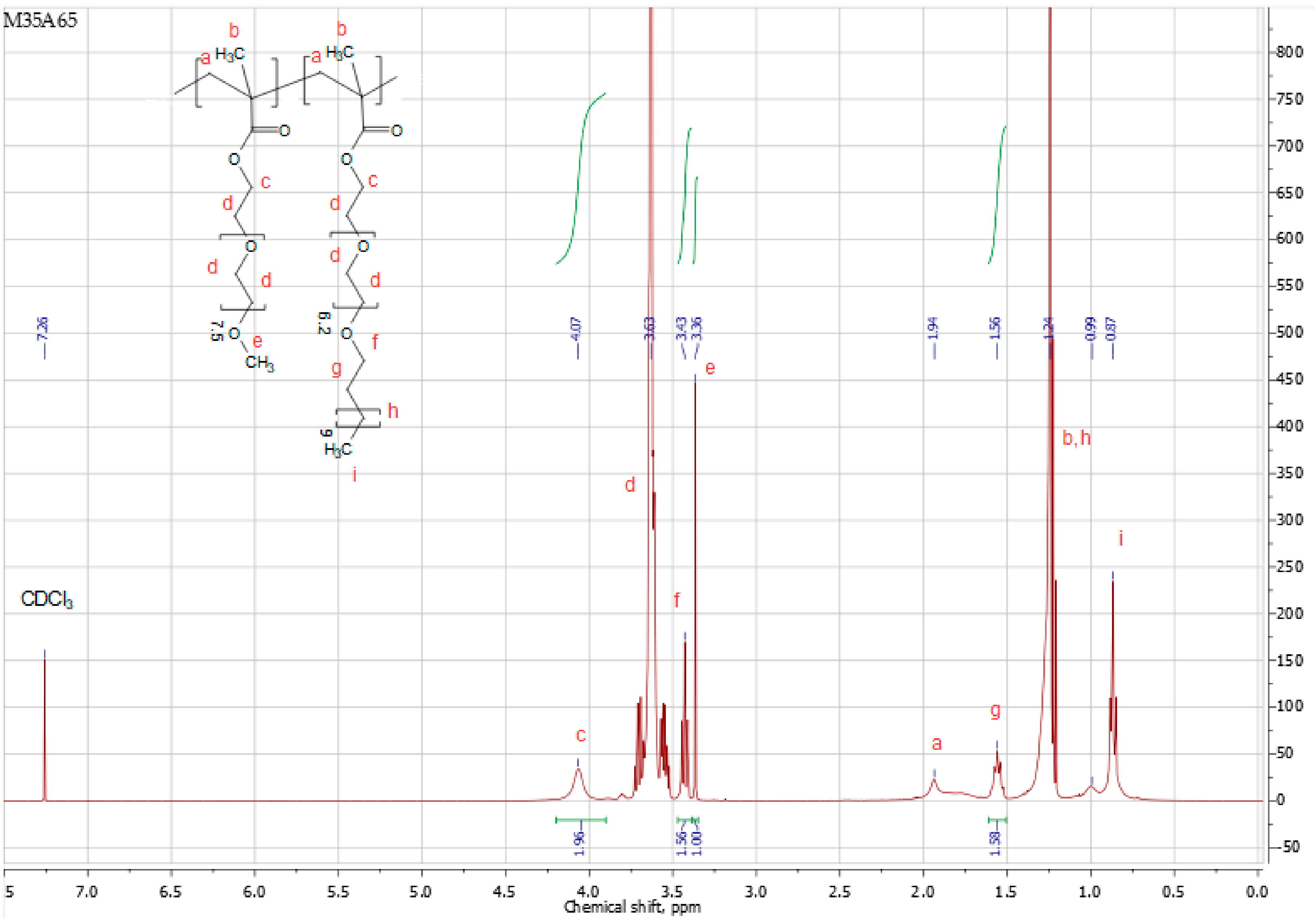

Figure S1. ^{1}H NMR spectrum of M35A65 copolymer in $CDCl_3$

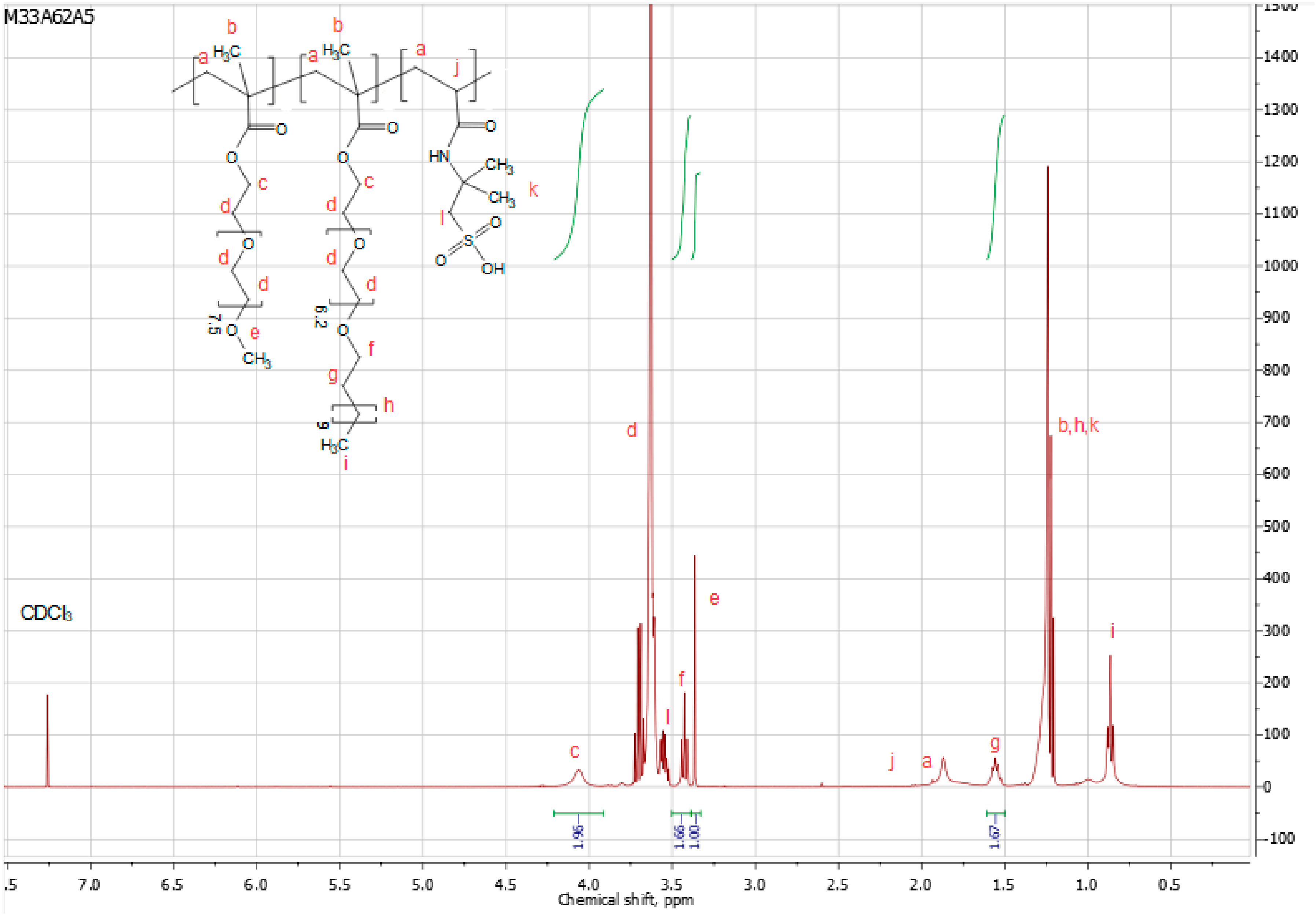

Figure S2. ^{1}H NMR spectrum of M33A62A5 copolymer in $CDCl_3$

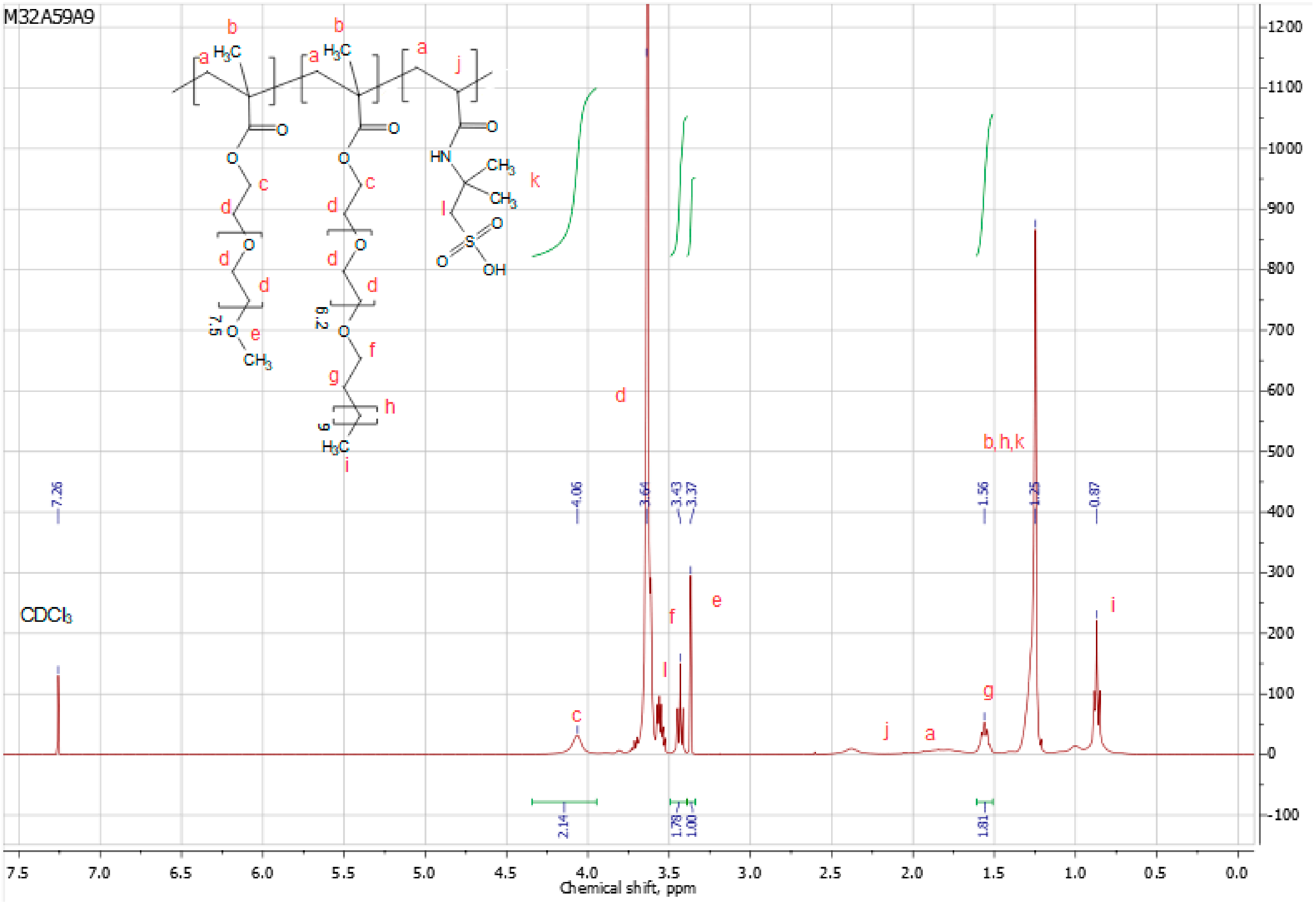

Figure S3. ^{1}H NMR spectrum of M32A59A9 copolymer in $CDCl_3$

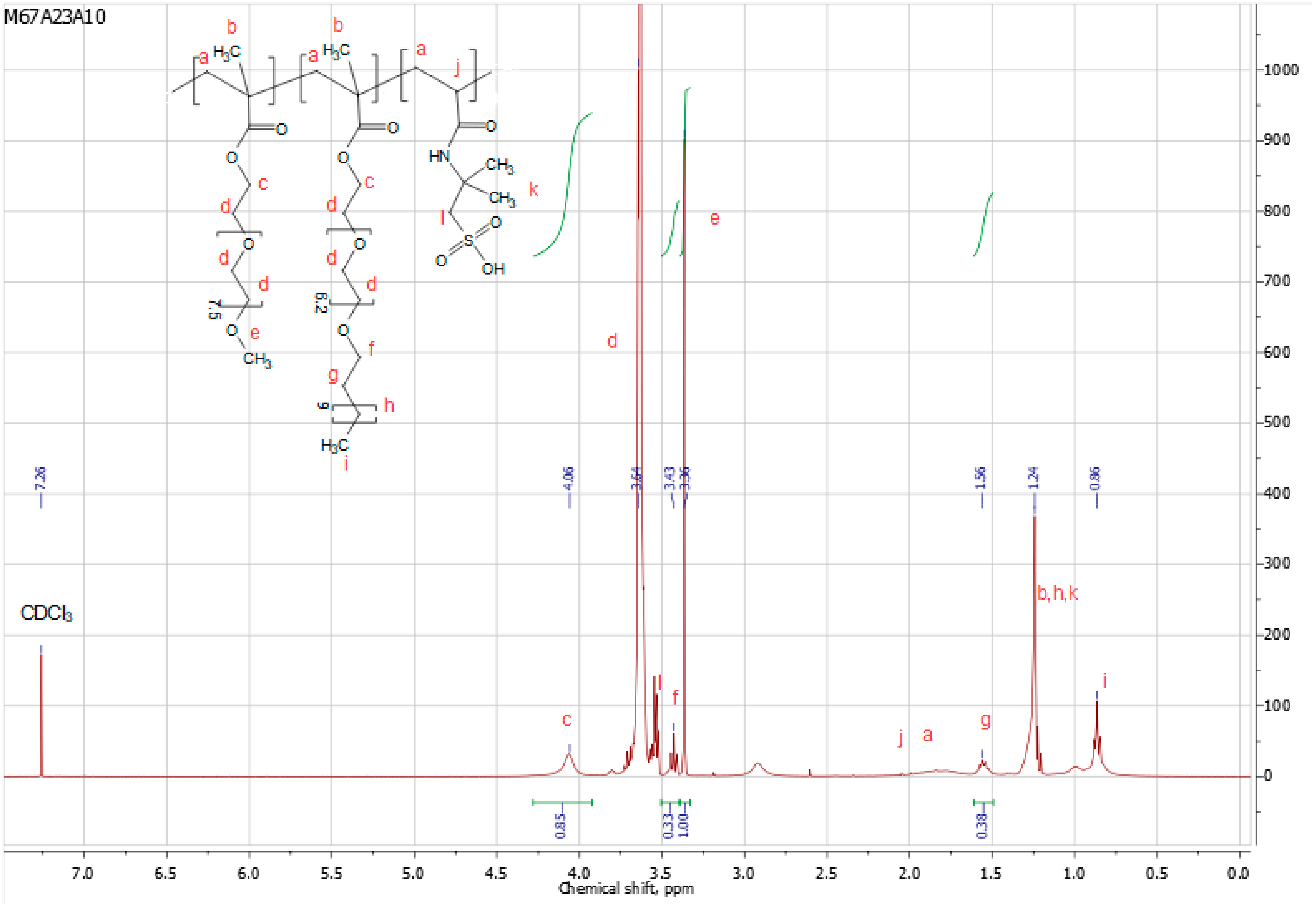

Figure S4. ^{1}H NMR spectrum of M67A23A10 copolymer in $CDCl_3$

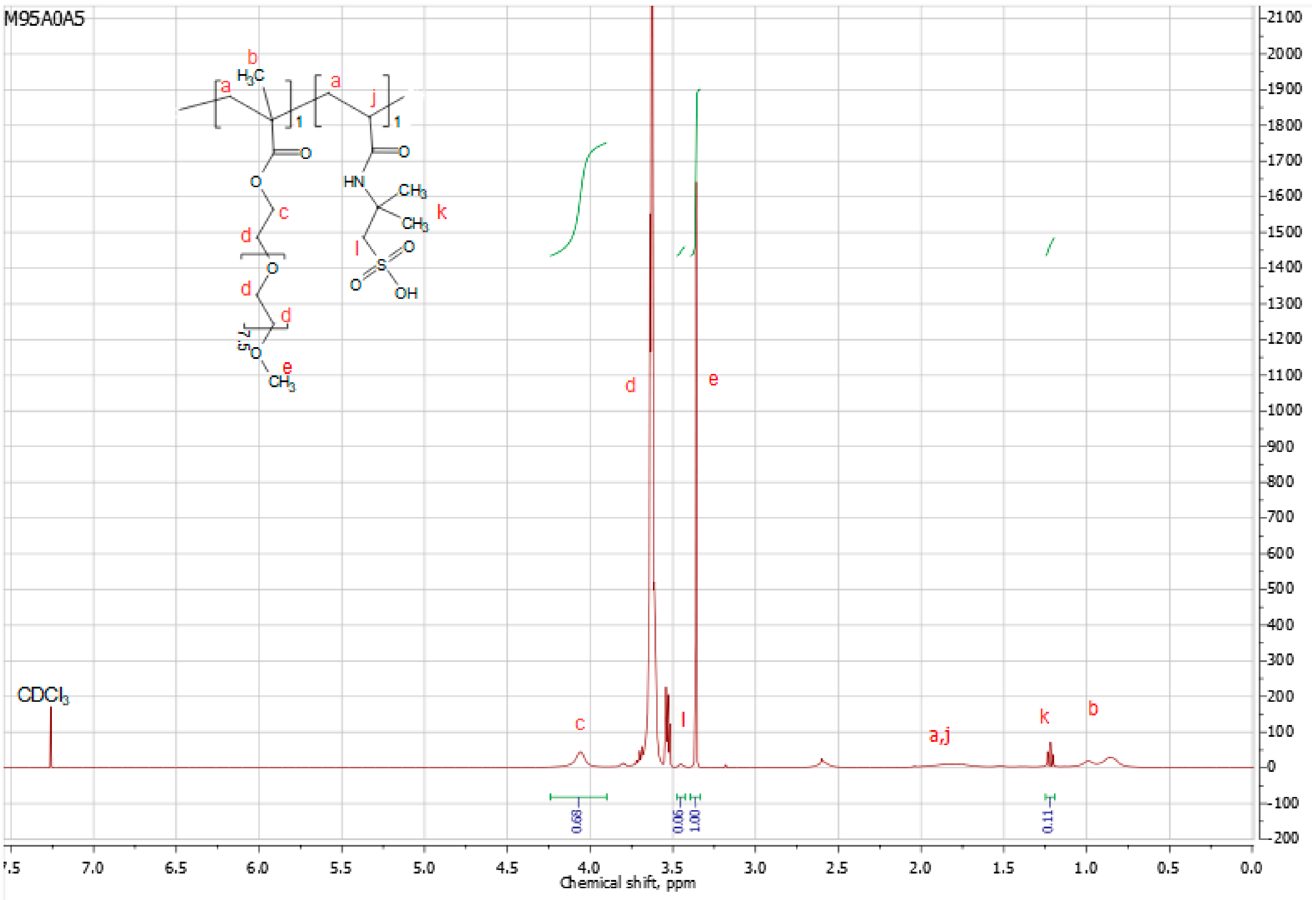

Figure S5. ¹H NMR spectrum of M95A0A5 copolymer in $CDCl_3$

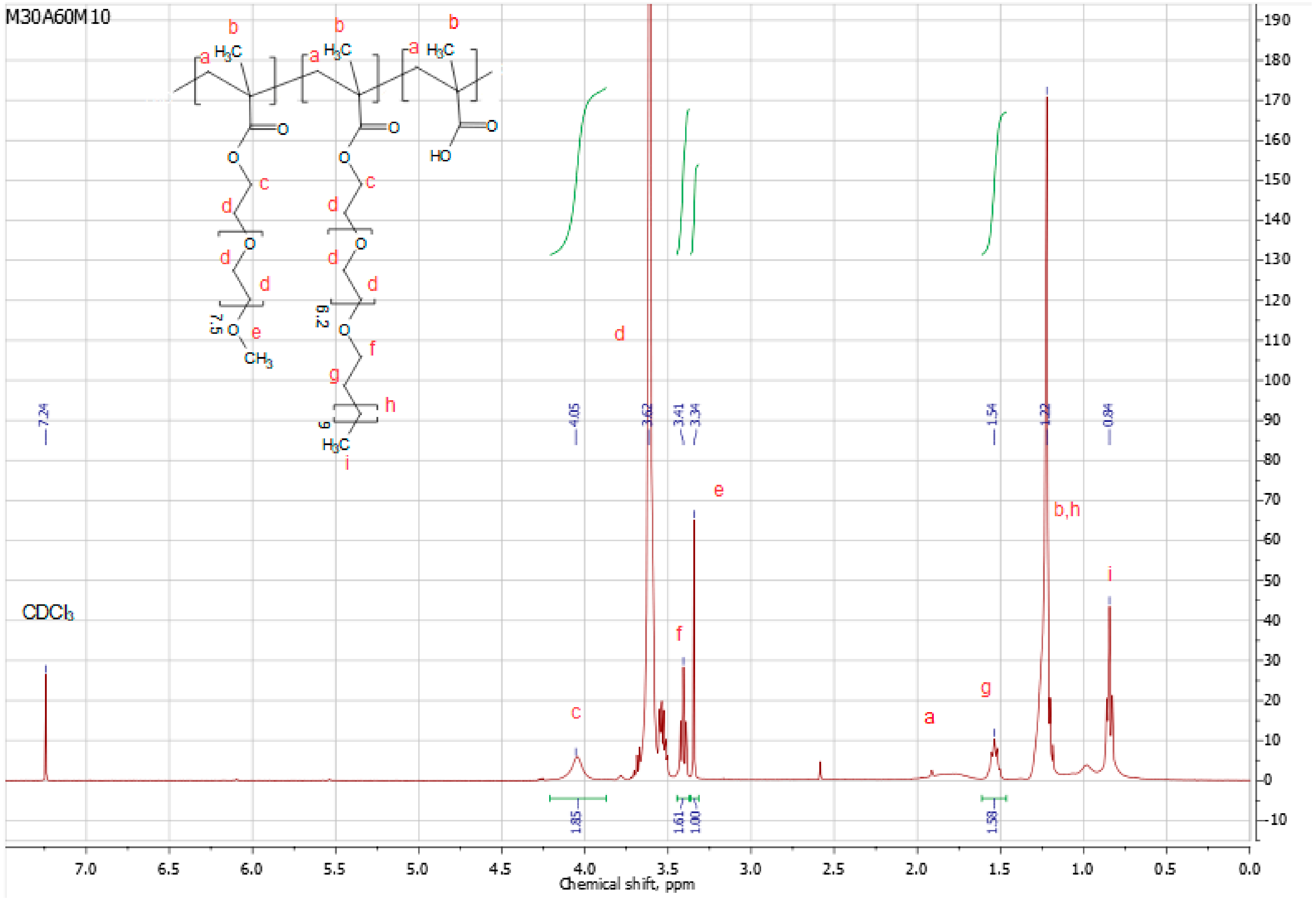

Figure S6. ^{1}H NMR spectrum of M30A60M10 copolymer in $CDCl_3$

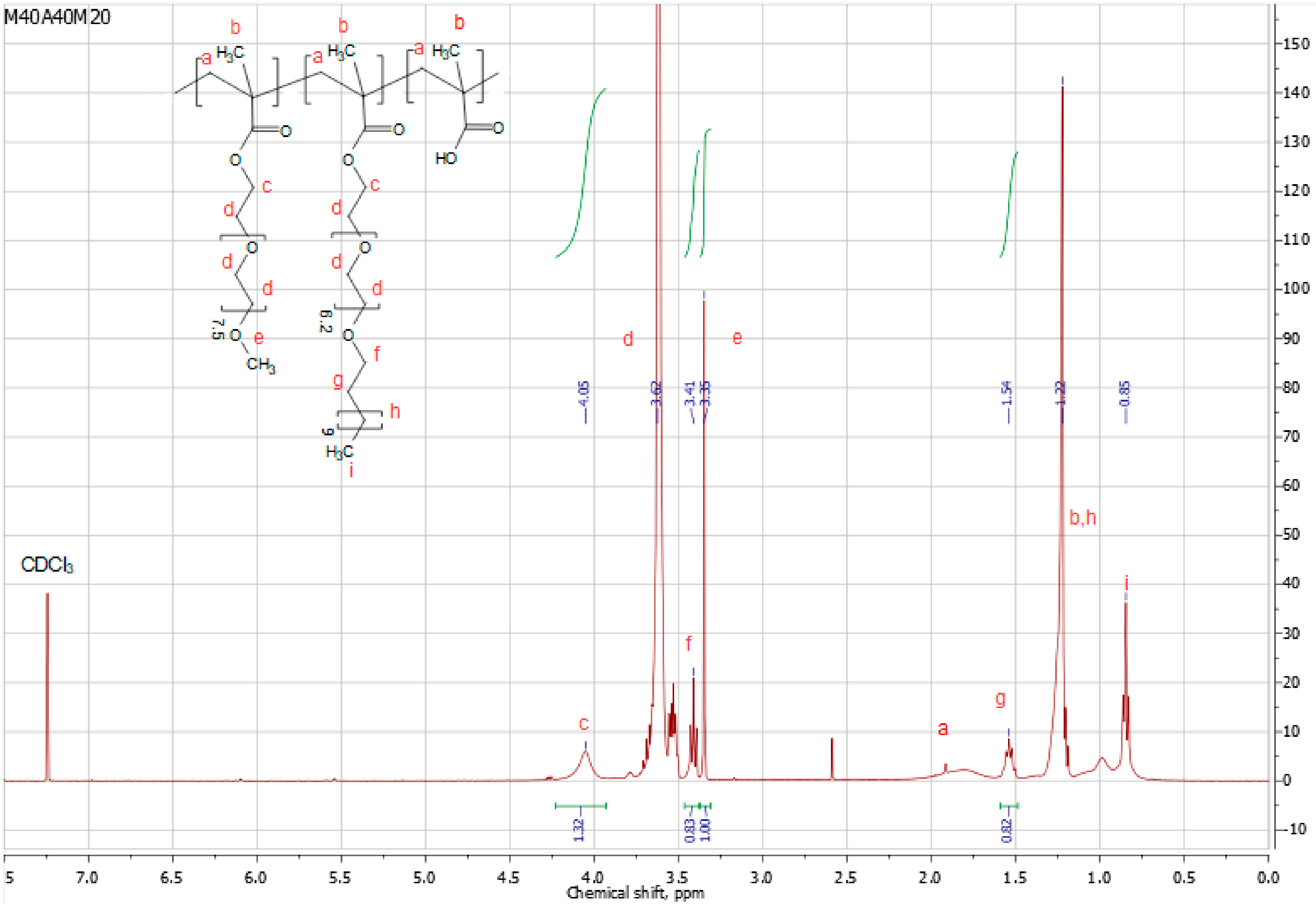

Figure S7. ^{1}H NMR spectrum of M40A40M20 copolymer in $CDCl_3$

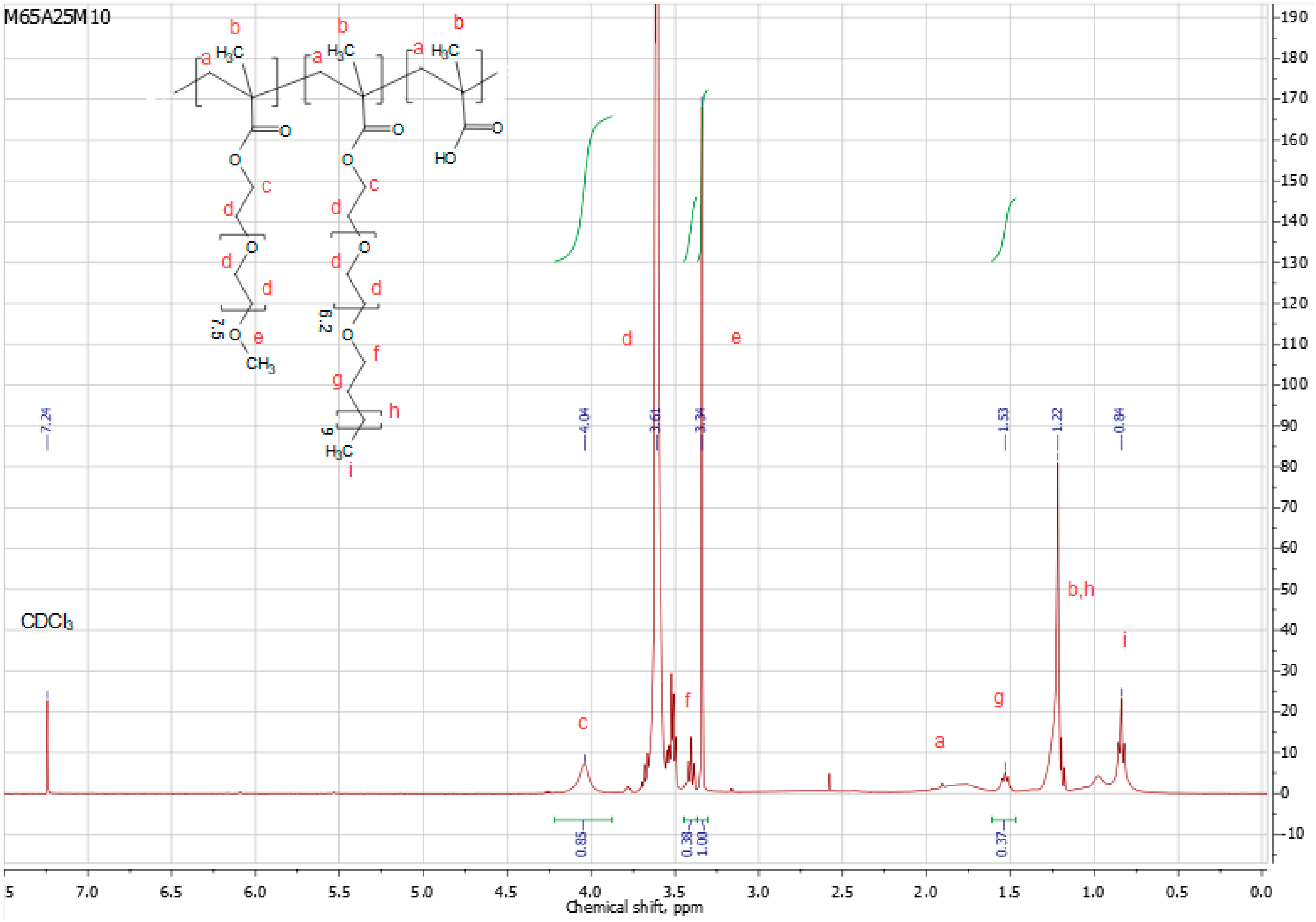

Figure S8. ^{1}H NMR spectrum of M65A25M10 copolymer in $CDCl_3$
